# Association of Types of Life Events with Depressive Symptoms among Puerto Rican Youth

**DOI:** 10.1371/journal.pone.0164852

**Published:** 2016-10-27

**Authors:** Graciela Jaschek, Olivia D. Carter-Pokras, Xin He, Sunmin Lee, Glorisa Canino

**Affiliations:** 1 Department of Epidemiology and Biostatistics, University of Maryland School of Public Health, College Park, Maryland, United States of America; 2 Behavioral Sciences Research Institute, University of Puerto Rico, San Juan, Puerto Rico, United States of America; Maastricht Universitair Medisch Centrum+, NETHERLANDS

## Abstract

The main objective of this study was to examine the association between four types of adverse life events (family environment, separation, social adversity, and death) and the development of depressive symptoms among Puerto Rican youth. This was a secondary analysis using three waves (2000–2004) of interview data from the Boricua Youth Study of 10–13 year old Puerto Rican youth residing in New York and Puerto Rico with no depressive symptoms at baseline (n = 977). Depressive symptoms increased with an increase in social adversity, separation, death, and death events. Youth support from parents was a significant protective factor for all adverse events and parent coping was a protective factor in social adversity events. Relying on standard diagnostic tools is ideal to identify youth meeting the criteria for a diagnosis of depression but not useful to detect youth who present with subclinical levels of depression. Youth with sub-clinical levels of depression will not get treated and are at increased risk of developing depression later in life. Adverse life events are potentially relevant to use in conjunction with other screening tools to identify Puerto Rican youth who have subclinical depression and are at risk of developing depression in later adolescence.

## Introduction

Depression is a rare psychiatric disorder in childhood but becomes one of the most common disorders in adolescence [1] with trajectories during this transition that are still not well understood [2]. Research on Latino youth mental health remains relatively rare [3], and little is known about youth depression among Latino subgroups [4]. Our interest in Latino youth was fueled by the rapid increase of Latinos younger than 18 in the U.S between 1993 and 2013 (107% compared to 11% for the general population) [5]. We focused on Puerto Ricans who are the second largest group of Latinos living in the U.S. (4.6 million), representing 9% of the Latino population [6]. Puerto Ricans are more likely to possess social characteristics that have been shown to threaten family stability, and place youth at an increased risk for developing depression compared to other Latino subgroups [7]. Youth living within Puerto Rican families are more likely to face social disruption (e.g., high alcohol consumption, divorce rates, criminality, and school dropout rates) and a lower socioeconomic status [8]. Social disruption has been shown to be associated with early onset depression in adolescence [9, 10] which in turn often results in serious social and health consequences later in life [11]. Adolescent depression may progress to severe chronic depression and overall poorer psychosocial outcomes in young adulthood [12], and higher recurrence rates in adulthood (40–60% recurrence) [13]. The effects of early onset depression may also be intensified by increases in exposure to potentially disruptive life events as adolescents grow and gain more autonomy [14–18].

Life events have been examined through the use of checklists for almost five decades (e.g., Life Events Checklist, Life Experience Survey, Stressful Life Events Schedule) [19–21]. However, checklists are conditional on the nature of the research being conducted and present a further challenge by the choice, multiplicity and character of events [22]. For example, early research based on the Schedule of Recent Experiences focused on the effects of events commonly encountered in normal life [23], while critics argued that life events have different weights and that their personal significance may result in more stress for some individuals but not for others [24]. Later research broadened to focus on stressful life events and their differential impact on mental health [25] given the role of stress in the etiology of mental illness and specifically depression [26]. Certain types of life events (i.e., loss of family members or friends, family history of offending and mental disorder, recent domestic and family problems) in populations such as incarcerated adults [27], the homeless [28], and adults with alcohol dependence [29], have been found to be more strongly associated with outcomes such as depression, suicide attempts and homelessness than other life events. Grouping life events in related categories may provide clues about how each type of life events affect Puerto Rican youth living in two different contexts (the Bronx, New York and San Juan, Puerto Rico). In addition, a more nuanced understanding of the effects that specific types of life events have on the development of depressive symptoms may serve as a guide to develop more effective screening tools and interventions. In this study we use multiple categories to group congruent life events (e.g., loss, death, autonomy, deviance, accident/illness, health, relocation events) [30, 31]. Although most individuals will be exposed to traumatic events and losses throughout their lives, only some will develop depression while the majority will recover and move forward [32]. The mechanisms by which stressful events affect individuals differently are not fully understood [33] but studies show that resilient individuals overcome challenges by mobilizing personal and social resources to cope with stress and adversity [34].

Research among adolescents and adults shows a significant association between stressful life events and depression or depressive symptoms [35, 36]. However, many depressed youth do not experience a stressful life event prior to their depression and not all youth who experience stressful or adverse life events become depressed. Successful adaptation to stress depends on the environment and the resources available to the youth to cope with the stressful life events [37]. The choice and/or the efficiency of coping strategies used in response to stressors will vary with each individual [38], and that choice will determine how well the individual copes with a situation or is affected by stress [39–41]. Ongoing support from family members, peers and other adults is known to buffer individuals from the psychological consequences of stressors [42, 43]. Social support alleviates psychological stress triggered by life events and has been identified as a protective factor for depression [44, 45]. The most influential theoretical perspective on social support states that the perception of available support and the supportive actions of others reduce the effects of stressful life events on health [46]. Place of residence is a third factor that can indirectly influence the development of depression through psychosocial processes such as personal control, social support, and stress [47]. Studies show that migrant children have a higher prevalence of internalizing disorders (depression and anxiety) than native children [48]. Racism, prejudice, and discrimination in the contextual environment may drive minority families to function in denser urban environments that offer minimal resources and opportunities [49] and exposes individuals to poverty, low education, low employment, and low social support [50].

The main objective of this study is to identify and compare types of life events that are significantly associated with depressive symptoms among 10–13 year old Puerto Rican youth. This study seeks to examine different types of life events that lead families to physical, psychological or interpersonal crises, as postulated in Hill’s Theory of Family Stress [51]. We aim to find what types of events have a greater impact on adolescent mental health that results in a greater number of depressive symptoms. Our hypothesis is that certain types of life events have a greater developmental impact and will be more strongly associated with depressive symptoms. We also test place of residence, youth coping and social support as effect modifiers [43, 52–54]. The analytic sample includes children living in the majority context of San Juan, Puerto Rico, and the minority context of the Bronx, New York. Comparing Puerto Rican children in these two contexts will expand our understanding of the effect of place on depressive symptoms.

## Materials and Methods

This study uses data collected in three annual waves between summer 2000 and fall 2004 of the Boricua Youth Study (BYS) [55]. The BYS is a longitudinal study of child and parent/caregiver data on psychiatric disorders that uses multistage probability samples to represent 5–13 year old Puerto Rican children living in the South Bronx, New York and San Juan, Puerto Rico according to the 2000 U.S. census. Eligible households included at least one 5 to 13 year old child residing in the household at the time of the census, with at least a parent living in the household who was of Puerto Rican background. Up to three children were selected among households with multiple children. Trained lay interviewers conducted home interviews separately but concurrently with children and parents/caregivers in Spanish or English based on language preference. In the following paragraphs we include an explanation of reporters for each measure. The BYS includes data on family socio-demographic characteristics, child psychopathology and other risk factors for mental disorders. Parents provided signed informed consent, while children 7 or older provided signed informed assent. The attrition rate was 7.95% at wave 2, and 11.88% at wave 3. For a more detailed description of the study’s background, design and methods refer to work conducted by Bird et al. (2006). Institutional Review Board approval for this project was obtained from the University of Puerto Rico and the University of Maryland.

The analytic sample included 977 Puerto Rican children 10–13 years old who met the following conditions: 1) no depressive symptoms at wave 1; 2) participated in both waves 1 and 2; 3) had complete life events data for both waves 1 and 2; and 4) had complete depressive symptoms data at waves 2 and 3. We excluded children with depressive symptoms at wave 1 (n = 155) in order to establish a clear temporal sequence between life events at wave 1 and the development of depressive symptoms at waves2, and then life events at wave 2 and the development of depressive symptoms at wave 3 respectively. We also excluded youth missing life events information at wave 1 and wave 2 (n = 5). Children 5–9 years of age were excluded from the analysis because the reliability of the instrument measuring psychiatric disorders in this population is poor [56].

### Depressive Symptoms

Depressive symptoms (reported at waves 2 and 3 by both parents and children) was the count of symptoms experienced in the past 12 months. We chose to use the DISC Predictive Scale (DPS) which asks a series of 9 questions of all respondents and accurately predicts depression [57]. The DPS is derived from the National Institute of Mental Health Diagnostic Interview Schedule for Children, Version IV (DISC-IV) Major Depression Schedule [58] and has been recommended to be used as a standardized tool for quick evaluation of children and adolescent diagnoses. The DPS is a structured diagnostic instrument that includes ‘yes’ and ‘no’ responses which limit the level of interpretation and decision making by the interviewer and is ideal for large population-based studies. The DPS is based on the Diagnostic and Statistical Manual of Disorders (DSM-IV), administered to youths and parents by trained lay-interviewers. Depressive symptoms were a combined measure of positive responses to the 9 questions reported both by children and their primary parents/caregivers. We chose this combined measure to lessen reporter bias if only children reported on depressive symptoms. [58]. Lucas et al. (2001) calculated the reliability of the scale to be good (Cronbach’s α = 0.82). The English version of the DISC-IV is a commonly used instrument to ascertain a wide range of child and adolescent psychiatric diagnoses. DISC-IV is only one of four instruments that have been translated into Spanish and only one of two instruments with psychometric data available for U.S. Latinos [59]. The moderate test-retest reliability of the Spanish version was found to be similar to the English version (κ = 0.48) [60]. We used a combination of children and parent reports of depressive symptoms in order to obtain the most reliable information [60].

### Types of Life Events

The twenty life events used in this study were derived from a list of 46 items from the Life Events Checklist [19]. Life events in the past 12 months were self-reported by the youth at waves 1 and 2. The four types of life events used in this study (separation, death, family environment, social adversity) were adapted from the research on types of life events conducted by Grover et al. [61]. Types of life events are based on developmental models and were identified as potential predictors of psychopathology [62]. The types of events included: 1) separation events (move to a new home, start a new school, parents separate, parental divorce, you break up with a girl/boyfriend, a close friend moved far away, a family member went to jail2) death events (your pet died, a close friend died, a close family member died) 3) social adversity events (you were victim of a crime, you saw a crime, a family member was arrested, a family member has an alcohol/drug problem; and 4) family environment events (parents argue more, have a new brother/sister, have a new stepfather/stepmother, you got sick or injured, a family member has a mental or emotional problem, family member was sick or injured). Each type of life event was an aggregate of the youths’ positive responses to the life events included in that category.

### Socio-Demographic Variables

Socio-demographic variables included: age (integer in years), gender (reported by the interviewer), place of residence (San Juan, Puerto Rico; and South Bronx, New York as recorded by the interviewer in the Profile section), family composition (single or two biological/other parent family as reported by parents), per capita household income (calculated by dividing household income by the number of household residents as reported by parents), and parental education (less than high school, high school, more than high school as reported by parents). *Parent/caregiver received social support* was based on the count of positive responses by parents to three out of 15 items selected from the Parent Social Support scale originally developed by Thoits [38]. We calculated the internal consistency for three items that characterized received social support. The reliability coefficient was acceptable for the three items (Cronbach’s α = .70, at wave 1). The items focused on: “How often do you get together with family members you don't live with?” “How often do you attend family gatherings?”, “How often do family members help you take care of your kids?” Similar concepts on received social support can be found in the literature [63–65]. Responses ranged from 0 = never/once a year or less, 1 = several times a year but less than once a month, 2 = less than once a week but at least once a month, 3 = at least once a week for the three items. Only one item from the Parent Social Support scale measured *parent/caregiver perceived social support* and was selected for this study [38]. Responses by parents to the item ranged from 0 = never/once a year or less, to 3 = at least once a week: “In general how satisfied are you with the amount of support that you receive in your life.”

### Diagnosed Lifetime Maternal Depression

Diagnosed depression was self-reported by the mother based on the Family History Screen for Epidemiologic Studies (FHES) [66]. FHES shows good sensitivity (88.5%) and specificity (73.3%) for lifetime maternal depression. Aggregated positive responses to the Parent/Child Interaction scale originally developed by Loeber et. al. [67] measured *youth support from parents*. The scale has 12 items and responses were reported by youth informants. Reliability was calculated for this study and found to be good (Cronbach’s α = .74, at wave 1). Responses range from 0 = rarely or never and 1 = sometimes or often for all items. *Parent/caregiver coping* was a count variable of positive responses by parents to all questions in the Parental Coping scale. The scale was originally developed by Thoits [38], and consisted of eight items ranging from 0 = *almost never*, 1 = *sometimes*, to 2 = *often* for all items such as “Have you felt that you were unable to control the important things in your life?” (reverse coded) and “Have you successfully dealt with irritating life hassles?” The internal reliability of this scale was calculated for this study (Cronbach’s α = .70, at wave 1) and was found to be in the acceptable range [68]. *Youth coping* was derived from the Ways of Coping scale which was administered to youth informants and consisted of 5 items: “You do what you have to do to solve it. Would you say you do that?”, “You wait for the problem to go away by itself.”, “You go and play or do something else.”, “You talk to someone who can help you with the problem.”, “You try to see the good side of the problem.” Answers were recorded as 0 = *rarely or never* or 2 (recoded as a 1) = *sometimes or often*. The items on the scale were only measured at wave 1 and dichotomized (low = <3 items, high = ≥3 items) based on the mean (3.28 items). We calculated the reliability for the Ways of Coping scale and reliability was low (Cronbach’s α = .45, at wave 1). As a result of the low reliability, the variable was dropped from the analyses. Coping scales have been problematic as a result of disagreement about the classification, the number, and the types of coping [41, 69]. The measurement of coping has also been challenging and investigators have commented on inadequate component structures and low scale reliability [41, 70–72].

Univariate analyses were conducted to calculate descriptive statistics and to check for missing values, and outliers. Bivariate analyses were conducted to identify potential confounders for each type of life event: a variable was considered a confounder and was added to the final model when there was a 5% change in the regression coefficients. Confounders were screened using bivariate analysis and entered into the final models. We assessed the internal consistency of all study scales by calculating correlations between scale items in the full analytic sample using Cronbach’s alpha coefficient. Multicollinearity was tested using tolerance tests with the standard cutoff of 0.1 and tolerance values were found to be in the acceptable range between 0.97 and 0.99. Statistical interaction was tested for each type of life event by separately introducing an interaction term for each potential effect modifier (place of residence and social support). A log-linear Poisson model was used as a result of the overdispersion of negative answers to depressive symptoms. We examined the association between the types of life events (social adversity, separation, death, family environment) in the previous 12 months at wave 1 and depressive symptoms at wave 2, and types of life events at wave 2 and depressive symptoms at wave 3 among youth with no depressive symptoms at wave 1.

Selection of covariates and descriptive statistics were conducted using SAS Software Version 9.3, Copyright SAS Institute Inc. Weighted analyses were conducted with SUDAAN software (release 11, 2012) [73] to adjust standard errors for correlations resulting from the complex multistage sampling (observations nested within individuals, siblings nested within households, and households nested within census blocks). SUDAAN adjusts for intraclass correlations using a sandwich variance estimator with a one-level covariance structure at the PSU level. No separate variance components at the household and PSU levels were estimated. Sampling weights were used in all analyses to adjust for different probabilities of selection and to be representative of the age and gender distribution of the 2000 census. All p-values were considered significant at the ≤ 0.05 level.

## Results

Youth experienced a mean of 2.00 (SD = 1.93) life events, separation events ranged from 0 to 7 events with a mean of 0.92 (SD = 1.04), death events ranged from 0 to 4 events with a mean of 0.54 (SD = 0.70), social adversity life events ranged from 0 to 4 events with a mean of 0.24 (SD = 0.52), and family environment events ranged from 0 to 4 events with a mean of 0.31 (SD = 0.61). Table 1 shows weighted mean depressive symptoms at wave 2 for 10–13 year old youth without depressive symptoms at wave 1 by socio-demographic characteristics and types of life events at wave 1. Overall, a simple inspection shows that mean depressive symptoms were higher for youth who experienced one or more type of life events compared to youth who experienced no type of life event.

**Table 1:** Mean Depressive Symptoms (wave 2) by Socio-Demographic Characteristics (wave 1) and Type of Life Event (wave 1) for 10–13 Year Old Youth without Depressive Symptoms at Wave 1 (weighted).

			Social Adversity Events		Separation Events		Death Events		Family Environment Events	
	% Weighted	Total Sample	(0)	(1–4)	(0)	(1–7)	(0)	(1–3)	(0)	(1–6)
		(n = 1,044)	(n = 784)	(n = 260)	(n = 420)	(n = 624)	(n = 553)	(n = 491)	(n = 757)	(n = 287)
**Place of Residence**										
Bronx	83.5	2.8	2.6	3.6	2.5	3.0	2.5	2.9	2.6	3.3
Puerto Rico	16.6	2.5	2.1	3.6	2.0	3.0	2.6	3.2	2.3	3.0
**Gender**										
Female	48.9	2.7	2.3	3.8	2.3	2.9	2.6	3.1	2.5	3.1
Male	51.1	2.9	2.7	3.4	2.4	3.1	2.2	2.8	2.7	3.4
**Age Distribution**										
10	27.8	2.5	2.4	3.3	2.1	2.8	2.5	2.6	2.1	4.2
11	23.6	2.4	2.2	2.9	2.1	2.6	2.1	2.7	2.3	2.5
12	23.5	2.7	2.3	4.1	2.2	3.0	2.6	2.8	2.5	3.0
13	25.1	2.9	2.7	3.4	2.6	3.1	2.8	3.1	2.8	3.3
**Mother's Education**										
Less than High School	40.3	2.8	2.6	3.4	2.5	2.9	2.6	3.0	2.5	3.4
High School Diploma/GED	43.6	2.8	2.5	3.5	2.3	3.0	2.6	3.1	2.6	3.1
More than High School	16.1	2.7	2.2	3.9	2.3	3.0	3.7	2.9	2.5	3.0
**Family Composition (Parents Living in Household)**										
Two Biological/Other Parent	54.7	2.8	2.5	3.6	2.3	2.2	2.6	3.0	2.5	3.2
One Biological/Other Parent	45.3	2.8	2.5	3.6	2.4	3.0	2.5	3.1	2.6	3.3
**Diagnosed Maternal Depression**										
Yes	4.7	2.7	2.4	3.6	2.4	2.9	2.5	3.0	2.6	3.1
No	97.3	4.0	3.9	4.2	2.6	4.4	3.8	4.2	3.0	5.9

Table 2 is a list of life events used in the Boricua Youth Study by type of event. Although not shown here, 63.2% of youth experienced separation events (e.g., move to a new home, start a new school, parents separate) compared to 41.8% who experienced death events (e.g., your pet died, a close friend died), and 22.6% who experienced social adversity events (e.g., you were victim of a crime, you saw a crime).

**Table 2:** List of Life Events used by the Boricua Youth Study Categorized by Type of Life Events.

**Social Adversity Events:** During the last 12 months…
1. Were you the victim of a crime, a violent act, or assault?
2. Did you see a crime or accident where someone was mugged, hurt or killed?
3. Was someone in your family arrested?
4. Did a family member have a drug or alcohol problem (not including you)?
**Separation Events**: During the last 12 months…
1. Did you move to a new home (permanently, not a temporary residence that is not your home)?
2. Did you start going to a new school?
3. Did your parents separate?
4. Did your parents get divorced?
5. Did you break up with a girlfriend or boyfriend?
6. Did you lose a close friend not by death, or did a close friend move far away?
7. Did someone in your family go to jail?
**Death Events**: During the last 12 months…
1. Did a close friend die?
2. Did someone in the family that you loved a lot die?
3. Did a pet of yours die?
**Family Environment Events**: During the last 12 months…
1. Did your parents argue more than previously?
2. Did a new brother or sister arrive in your home (because one was born, or one moved into the household)?
3. Did a new stepmother or stepfather move into your house?
4. Did you get seriously sick or injured?
5. Did a family member (not including you) have a mental or emotional problem?
6. Was someone in your family seriously sick or injured?

Table 3 shows exponentiated regression coefficients for effect size but they need to be interpreted carefully: A unit increase in social adversity events [b = 0.72, exp(b) = 2.05, p<0.0001] results in a 105% increase in depressive symptoms; a unit increase in family events [b = 0.41, exp(b) = 1.507, p = 0.0037] results in a 50.7% increase in depressive symptoms; a unit increase in death events [b = 0.43, exp(b) = 1.537, p = 0.0033] results in a 53.7% increase in depressive symptoms; and, a unit increase in separation events [b = 0.28, exp(b) = 1.323, p = 0.0013] results in a 32.3% increase in depressive symptoms. Youth support from parents was a significant risk factor in the association between all types of events and depressive symptoms. Parent coping was a significant risk factor in the association between social adversity events and depressive symptoms. Youth support from parents (p = 0.0533), and place of residence (p = 0.9942) were tested as effect modifiers but were not found to modify any of the associations between types of life events and depressive symptoms.

**Table 3:** Log-Linear Regressions of Separation, Death, and Social Adversity Events in the Last 12 Months and Depressive Symptoms.

	Unadjusted Model			Adjusted Model		
	exp(b)	SE	p-value	exp(b)	SE	p-value
**Separation Events**^**1**^	1.32	0.08	**0.0013**	1.31	0.08	**0.0022**
Youth Support from Parents				0.90	0.04	**0.0020**
**Death Events**^**2**^	1.54	0.14	**0.0033**	1.54	0.14	**0.003**
Youth Support from Parents				0.89	0.04	**0.0017**
**Social Adversity Events**^**3**^	2.05	0.12	**<0.0001**	1.97	0.16	**<0.0001**
Youth Support from Parents				0.90	0.04	**0.0087**
Parent Coping				0.91	0.04	**0.0132**
**Family Events**^**4**^	1.51	0.14	**0.0037**	1.48	0.14	**0.0068**
Youth Support from Parents				0.90	0.04	**0.0038**

^1^Adjusted for place of residence and mother’s depression

^2^Adjusted for gender

^3^Adjusted for age

^4^Adjusted for place of residence and gender

## Discussion

Research consistently shows that the experience of stressful life events is significantly associated with an increase in depressive symptoms and the onset of major depression [74–77], and first episodes of depression are usually preceded by a major stressful event [77, 78]. The “kindling” hypothesis [79] states that the first occurrence of depression sensitizes the individual to subsequent life events, and certain types of life events (e.g., severe loss, interpersonal trauma) significantly increase the likelihood of developing depression [80–82]. However, other studies indicate that the relationship may not be unidirectional and report that adolescents who are depressed will report more stressful life events than adolescents who are not [83, 84]. We looked at depressive symptoms at the end of 12 months among adolescents who experienced any type of life events and had no prior depressive symptoms to establish a clear temporal sequence. Overall, our findings lend support to research that adopt a unidirectional model between life events and depressive symptoms. We found that the association between types of events (e.g., social adversity, death, family, and separation events) and depressive symptoms varies in strength suggesting that life events have different impacts. Although separation events were the most commonly reported events, social adversity events had a greater negative impact on psychological well-being. These results suggest that adolescents may benefit from screenings and interventions that focus on specific developmentally grounded types of life events. Tailoring new screening tools and interventions to prevent depression by incorporating types of life events may prove to be a successful strategy. The different types of life events may provide clues to the adolescent’s environment that should be taken into account [85]. For example, a review of school-wide interventions for the prevention of depression concludes that universal school-wide interventions may not be efficacious or efficient because they focus on the individual without looking at the environmental changes that are needed to prevent the development of depression [86]. In addition, some studies have shown that alternative interventions may have different clinical utility in depression prevention [87].

Our study found that youth support from parents was a significant risk factor in the association between social adversity, separation, death, family events and depressive symptoms. These results support the literature that shows that family and personal resources, and in particular parent’s social support promote youth mental health [88]. Our results are consistent with prospective studies of the diathesis-stress model indicating that parental support does not moderate the development of depressive symptoms [89, 90]. Youth support from parents, and place of residence were tested as effect modifiers but were not found to moderate the association between any of the four types of life events examined and development of depressive symptoms. Puerto Ricans are U.S. citizens and can move back and forth between mainland and the island without legal obstacles [91]. Starting in the 1960s a circular pattern of immigration and emigration between the island and mainland emerged and although it is hard to estimate the proportion of Puerto Ricans who move back and forth, the movement of people has become a key characteristic of Puerto Rican culture [92]. According to the 2009 American Community Survey, fewer than half (47%) of Hispanics of Puerto Rican origin lived on the island [91]. Puerto Ricans have a strong transnational identity with distinct cultural, social, and political characteristics that transcend place of residence and may explain our null findings for place of residence as a risk factor and as a moderator.

This study includes several strengths and limitations that may guide future studies. A strength of this study was that depressive symptoms are assessed with a measure that has been found to be reliable and valid (DISC-IV) [58]. Second, to our knowledge there are no other longitudinal studies of the association between types of life events and depressive symptoms among Puerto Rican youth. Lastly, examining different types of life events provides an opportunity to understand how each type of life event impacts depressive symptoms [36, 93].

There are several limitations. The first limitation of this study is that although the reliability of depressive symptoms was found to be reasonable in this study (κ = 0.64) [58], depressive symptoms do not constitute a diagnosis of depression. Many adolescents have depressive symptoms that tend to diminish between adolescence and young adulthood but some youth show a persistent high level of depressive symptoms throughout adolescence and have a higher probability to develop depression in young adulthood [94]. In our study we do not identify the trajectories of depressive symptoms into late adolescence and may be overestimating the effect of types of life events overtime. However, our interest lies in being able to identify youth at risk of developing depressive symptoms and not necessarily youth who develop depression. Second, it is difficult to establish causality between types of life events and depressive symptoms because types of events and depressive symptoms may be dependent events and each could modify the reporting of the other thus inflating the association [95]. Our study supports the hypothesis that the association between types of life events and depressive symptoms is unidirectional making dependency between events and depressive symptoms less consequential. Third, symptoms of depression are transient, particularly in the absence of a depressive disorder. Therefore, it is uncertain whether a type of life event will have a sustained effect over a period of a year. Youth were asked about the types of life events they experienced over the preceding 12 month period and a positive answer was counted as an event but the exact timing of the event was unknown. We found that some types of life events are not discrete and the event develops or its consequences are felt over a long period of time. For example, if we examine separation events they include parents separating or parents divorcing, which are not discrete events. In these cases, the exact timing of the event may not be important because a separation or a divorce may take several months to be resolved. Fourth, recall of certain types of events may decrease or increase at different rates over time, and if recall is dependent on the type of life event there may be reporting bias. The association between certain types of life events and depressive symptoms may be stronger or weaker depending on the type of event [96, 97]. Fifth, we looked at four types of life events but we do not know which individuals experience more than one type of life event and in what combination [98]. Youth may experience any combination of positive and negative events within a type of life event and the nature of the combination of events may potentially trigger or protect from depressive symptoms. For example if we consider death events, the death of a pet may be a good thing if the pet was vicious, and the death of a family member may be very stressful. Last, children excluded from the study had more depressive symptoms and experienced more types of life events. We excluded those children because we wanted to establish a clear temporal sequence among symptom-free youth. Children with a previous history of depressive symptoms would bias our results to show a stronger association between types of life events and depressive symptoms.

Future studies may examine how each single life event within types of life events (e.g., death of a family member, death of a close friend, death of a pet) may happen in a sequence (i.e., parents argue more, parents separate, parents divorce), how that sequence affects the development of depressive symptoms, and the association between each element in a sequence of life events and depressive symptoms. Another area of future research may examine different ways of categorizing life events such as a sequence of events and the possible mechanisms for developing depressive symptoms. Last, future studies should further explore which types of life events are significantly associated with a first episode of depression and recurrence in later adolescence and young adulthood.

## Conclusions

This study shows that certain types of life events are more strongly associated with the development of depressive symptoms possibly highlighting a stress-sensitization effect that results in more depressive reactions [99]. The identification of risk and protective factors that contribute to the early development of depressive symptoms is of great clinical importance since early onset depression has lifelong economic, social and occupational impact [100]. Certain types of life events may serve as optimal indicators for the development and magnitude of subsequent depressive symptoms. Application of standard diagnostic tools to identify youth with depression, will miss youth who present with sub-clinical levels of depression (do not meet the criteria for a diagnosis) [101]. Youth with sub-clinical levels of depression will not get treated and are at increased risk of developing depression later in life [102, 103]. Using symptoms from the DISC Predictive Scale for depression coupled with additional measures such as reports of life events may prove useful in predicting the course of depression among youth who are not yet identified as having a diagnosis of depression. This study shows that separation, family, death, and social adversity types of life events are useful predictors of depressive symptoms among 10–13 year old Puerto Rican youth and that youth support from parents and parent coping is a significant protective factor in the association between types of life events and depressive symptoms. These findings may be useful to consider when trying to identify youth at risk of developing depression.
